# Unstable twin in body-centered cubic tungsten nanocrystals

**DOI:** 10.1038/s41467-020-16349-8

**Published:** 2020-05-19

**Authors:** Xiang Wang, Jiangwei Wang, Yang He, Chongmin Wang, Li Zhong, Scott X. Mao

**Affiliations:** 1 Department of Mechanical Engineering and Materials Science, University of Pittsburgh, Pittsburgh, Pennsylvania 15261 USA; 2 Environmental Molecular Sciences Laboratory, Pacific Northwest National Laboratory, Richland, Washington 99352 USA; 3 Present Address: Center of Electron Microscopy and State Key Laboratory of Silicon Materials, School of Materials Science and Engineering, Zhejiang University, 310027 Hangzhou, China

**Keywords:** Structural properties, Metals and alloys

## Abstract

Twinning is commonly activated in plastic deformation of low stacking-fault face-centered cubic (Fcc) metals but rarely found in body-centered cubic (Bcc) metals under room temperature and slow strain rates. Here, by conducting in situ transmission electron microscopy (TEM) at atomic scale, we discover that, in stark contrast to those in most Fcc metals, a majority of deformation twins in Bcc metals are unstable and undergo spontaneously detwinning upon unloading. Such unexpected instability of Bcc twins is found to be closely related to the prevalence of the inclined twin boundaries—a peculiar structure where twin boundaries are not parallel to the twinning plane, and the degree of instability is in direct proportion to the fraction of the inclined twin boundary. This work provides significant insights into the structure and stability of deformation twins in Bcc metals.

## Abstract (web summary)

Body-centred cubic metals rarely show twinning during deformation. Here, the authors use high resolution transmission electron microscopy to show tungsten, a body-centred cubic metal, spontaneously undergoes detwinning when unloaded.

## Introduction

Twinning as a common deformation mode plays a significant role in the mechanical properties of materials^1^. Twin boundaries can not only act as an effective barrier to hinder dislocation motion and accumulate dislocations but also provide the motion path and nucleation sites for dislocations, contributing to the excellent mechanical properties without the traditional trade-off between strength and ductility^2–4^. An ideal coherent twin boundary (CTB) with a coincidence interface is considered as a low-energy boundary with excellent thermal stability^3,5^, which provides massive space for property adjustments. Understanding the twinning ability and the stability of the twin structure is critical for engineering nanotwins toward advanced materials design.

Body-centered cubic (Bcc) metals, such as ferritic steels, are widely used as structural materials and magnetic functional materials. Different from metals with closed-packed structures such as face-centered cubic (Fcc), Bcc metals show poor twinning ability due to their high stacking faults energy^6,7^. As a result, dislocation plasticity normally dominates the plastic deformation of Bcc metals, whereas deformation twinning is only activated under extreme conditions, e.g., high stress, high strain rate, and low temperatures^8^. Recently, twinning-dominated plasticity in nanoscale Bcc metals was discovered by in-situ transmission electron microscope (TEM) experiment^9,10^, offering a good opportunity to directly study the twinning process and the thermodynamic stability of the twin that associate with the structures and energies of twin boundaries, which remains largely unclear in Bcc metals.

Here, by performing atomistic in-situ TEM study, we reveal that the twin stability and the detwinning process in Bcc tungsten (W) are strongly dependent on the type of twin boundary. Specifically, the stability of deformation twins in bcc metals is found to be controlled by a unique interface structure of the inclined twin boundary. Quantitative analysis demonstrates that the high energy of the inclined twin boundary contributes significantly to the driving force for detwinning and twins containing a high proportion of inclined twin boundaries show higher self-detwinning rates. Our work reveals the underlying mechanism of unstable deformation twins in Bcc metals and provides deep understanding of the (de)twinning behaviors, which is significant for the design and processing of twin structures in Bcc metals. Moreover, the spontaneous detwinning associated with the unstable twin brings possibilities to develop advanced materials with prominent pseudoelasticity and self-healing effect.

## Results

### Spontaneous detwinning

**Fig. 1:**
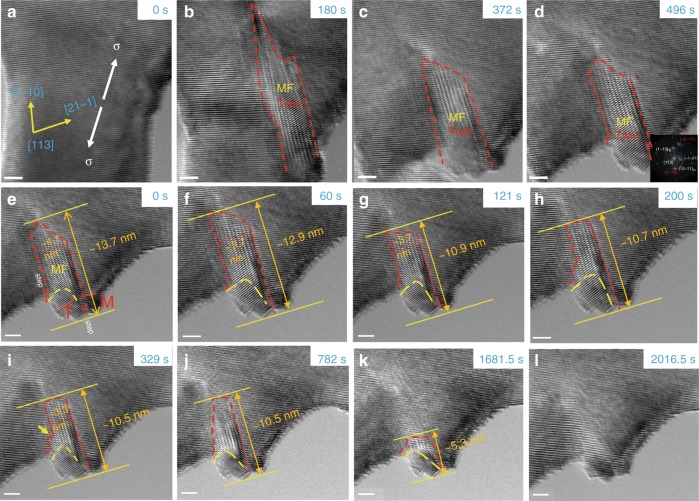
The spontaneous detwinning in the deformation twin containing ~70% Moiré fringes region. **a**–**d** The formation of the deformation twin under tension. **e**–**l** Spontaneous detwinning process upon unloading. Scale bar, 2 nm.

High-energy twin boundaries are generally deemed to reduce the stability of deformation twins. When a W single crystal was loaded as shown in Fig. 1a, a deformation twin was formed near the edge of the pillar. It is worth noting a large portion of the twinned region was filled with Moiré fringes (MF) (Fig. 1b, c). The MF is formed by overlapping of the twinned and matrix lattices^11^ as is proved by the diffraction analysis of the MF pattern shown in Supplementary Fig. 1A, indicating that the twin has not fully propagated along the crystal thickness direction. Therefore, there should exist an inclined twin boundary, which is nonparallel to the twinning plane between the twin and matrix in the overlapped region. This inclined twin boundary is fundamentally different from the traditional twin boundary–CTB and the MF region should coincide with the projection of this inclined twin boundary in the current viewing direction. Finally, a deformation twin with ~70% MF region was formed after the fracture of the pillar, whereas no contact existed between two fractured crystals after 496 s (Fig. 1d), excluding any loading effect on the following structural changes. Subsequently, a peculiar phenomenon, spontaneous detwinning of this twin occurred and proceeded steadily, as shown in Fig. 1e–l. The reduction of the MF region indicates the gradual retraction of the inclined twin boundary and the transformation from bulgy to flat-shaped twin boundaries is supposed to lower the interface energy^12^ (Fig. 1e–g). The length of the twin decreased quickly from 13.7 nm to 10.9 nm before 121 s (Fig. 1g) and then changed slowly when the size of inclined twin boundary has decreased significantly (Fig. 1h–j). The twin tip as the vertical front of the twin spontaneously retracted towards the bottom surface and the twin vanished quickly when the twin became very small and close to the surface (Fig. 1k, l). Detailed detwinning process could be found in Supplementary Movie 1.

Steps (marked in Fig. 1e) were often found on the inclined twin boundaries, which were regarded as the trace of the gliding of twinning partials on the CTB^13^. These steps disappeared during the detwinning (Fig. 1f), indicating that the reverse motion of twinning partials along CTB contributed partly to the self-detwinning process. Some parts of the inclined twin boundary moved relatively faster than others, as marked by the yellow arrow in Fig. 1e, which may result from the structural inhomogeneity along the inclined twin boundary. The detwinning process eliminated the twinned structure and strain, recovering the perfect lattice (Fig. 1l).

Electron beam irradiation is suspected to affect the twin boundary structures and migration of the twin boundaries^14,15^. To exclude this effect, beam-blanked experiments were carried out (see Supplementary Fig. 2). The retraction of the MF region still occurred and the twin disappeared after 40 min without the continuous electron beam exposure, implying that the observed detwinning process is intrinsic. Abundant of molecular dynamics (MD) simulations show that the driving force for detwinning during unloading in Bcc nanowires is attributed to the large surface energy difference between the {110} and {100} facets^16,17^. Here, our experiments show that spontaneous detwinning we discovered was very likely driven by the high energy of the inclined twin boundary rather than external effects.

### Profound differences in the structure of twin boundaries in Bcc tungsten and Fcc silver

**Fig. 2:**
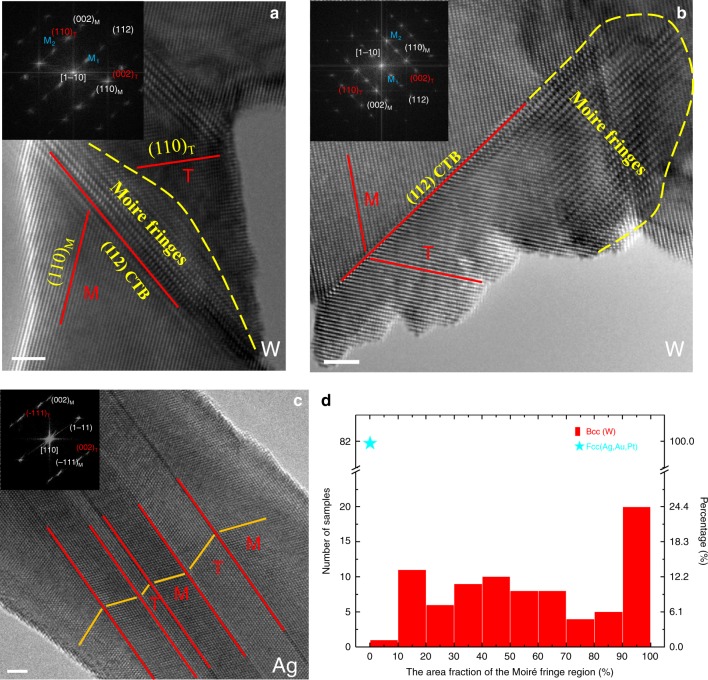
Representative deformation twins in body-centered cubic metals and face-centered cubic metals. **a** Deformation twin with the Moiré fringe near coherent twin boundary in tungsten. **b** Deformation twin with the Moiré fringes near the incoherent twin boundary (the twin head) in tungsten. **c** Typical deformation twin in silver. Fast Fourier transform patterns proving the twin structure are inserted in the figure. **d** The sample percentage of the area fraction of the Moiré fringes in deformation twins in tungsten and face-centered cubic metals (Ag, Pt, Au). Scale bar in **a**–**c** is 2 nm.

Interestingly, the existence of inclined twin boundaries in deformation twins is a common phenomenon in tungsten. Figure 2 shows representative deformation twins in Bcc (tungsten-W) and Fcc metals (silver, Ag) when both of them were viewed along the <110> direction parallel to the twin plane (i.e., CTB). Compared with the sharp twin boundaries in Ag (Fig. 2c), there exist a lot of inclined twin boundaries (see the MF patterns) near the CTB and the twin tip in W (Fig. 2a, b, respectively). The diffraction analyses of the Moiré fringe patterns in Fig. 2a, b are shown in Supplementary Fig. 1B, C. The MF patterns usually formed during twinning process under loading. Examples of MF forming under compression and tension can be found in Supplementary Fig. 3 and Supplementary Movies 2 and 3. The profile of the MF region is usually flexuous and irregular, implying that the slope of the inclined twin boundary is diversified. As a result, the detailed structure of this inclined interface cannot be determined directly by the projection view provided by the TEM image. The detailed three-dimensional (3D) structure of the inclined twin boundary needs to be studied by future advanced experiments and simulations. However, as a non-CTB, strong lattice distortion is deemed to exist near the inclined twin boundary, giving rise to higher strain energy compared with the CTB^18^. In addition, the shear strain within the matrix right in front of the twin tip provides an additional driving force for the detwinning.

Statistical analysis shows that >90% of the twins found in Bcc W possess the inclined twin boundaries (Fig. 1d). Moreover, the area fractions of MF region in deformation twins are also calculated (see Supplementary Fig. 4 for details). Most of the twins with over 90% MF counted here were captured during loading. By contrast, no MF formed in Ag crystal and the CTB is very sharp. The twin tip is usually bounded by an incoherent twin boundary parallel to the {112} plane, where the periodic stacking of twinning partials could lead to zero macro-strain^19,20^ (e.g., Supplementary Fig. 5A in Pt), and thus remarkably reduce the extra energy cost over the CTB energy^21^. Representative deformation twins in platinum and gold can be found in Supplementary Fig. 5. Admittedly, some distorted twin boundaries with MF were occasionally found in Fcc metals with low stacking-fault energies, which especially underwent severe plastic deformation, which might be induced by the operation of the pole mechanism that is hardly activated in Bcc metals ^1^.

### The proportion of the inclined twin boundary on detwinning

**Fig. 3:**
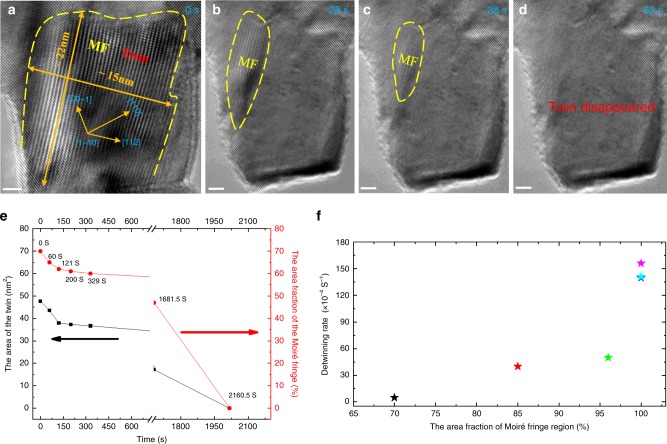
Dependence of the twin instability on the portion of the inclined twin boundary. **a**–**d** Detwinning in the twin with full Moiré fringes and ~15 nm thickness. **e** Development of the twin and the area fraction of the Moiré fringes with the detwinning time. **f** The dependence of the average detwinning rate on the area fraction of the Moiré fringes region. Scale bar in **a**–**d** is 2 nm.

The proportion of the inclined twin boundary plays an important role in the instability of deformation twins and detwinning in W, and most captured spontaneous detwinning happened in twins with over 70% MF region. As shown in Fig. 3a–d, detwinning proceeded quickly in the twin enriched in MFs, especially in the first 28 s, and completed after 62 s. This trend was also observed in Fig. 1, where the detwinning rate (i.e., the rate of reduction in the twin area) kept decreasing with the shrinking area of the MF (Fig. 3e), implying that the instability of the twin in Bcc metals is closely associated with the proportion of inclined twin boundaries. It is noteworthy that the abnormal increase in the detwinning rate after 1681.5 s in Fig. 3e can be attributed to the increasing imaging force^22^ acting on the twinning dislocations as they approached the surface (Fig. 1g, h). Similarly, detwinning could be facilitated when the twin was very small and near the surface, such as the self-detwinning processes observed in tiny twins as shown in Supplementary Fig. 6.

To further quantify the effect of inclined twin boundary on the stability of twins, the dependence of the average detwinning rate on the inclined twin boundary in twins with similar sizes (2–10 nm) was investigated, as shown in Fig. 3. Here, the detwinning rate is defined as the average changing rate of the twin area. Clearly, the detwinning rate increases with the MF area fraction. When the percentage of MF region is lower than ~60%, the deformation twins kept stable without obvious spontaneous detwinning after 30 min (Supplementary Fig. 7). This indicated the instability of the twin structure increases with the proportion of inclined twin boundaries and the interface energy of the inclined twin boundary provides the main driving force for detwinning.

## Discussion

The thermodynamic instability of deformed structures is largely decided by the excess energy compared with the perfect structure. For deformation twins, the excess energy is mainly from the interface energy of twin boundaries and the shear strain induced by twinning. The shear strain also induces the reorientation of the single crystal leading to the surface energy difference between the pristine crystal and deformed counterpart^16^. These excess energies provide the driving force for detwinning. MD simulation indicated that high-energy interfaces rather than coherent twin boundaries could induce the detwinning in Bcc nanowires during unloading^17^, consistent with our experimental observation.

As shown in Supplementary Fig. 3, the formation of the inclined twin boundary in Bcc metals is associated with the twinning process. One possible reason is the proposed double-cross-slip-assisted twinning process^23^. In the current study, we note that the propagation of twinning partials is very slow, which could be another factor contributing to the formation of inclined twin boundaries. Compared with the high glide velocity of twinning partials in Fcc metals^24^, the lattice friction is very high for dislocation slip in Bcc metals, especially for screw dislocations^25–27^. Besides, the twinning partials are deemed to be formed by the dissociation of full screw dislocations^28^, which move slowly through a kink-pair mechanism in Bcc metals^29^. In addition, kinks like small steps in screw parts of the twinning partial may increase the lattice distortion near the inclined twin boundary and its complexity further. Moreover, twinning partials nucleated from the surface are subjected to higher resistance when propagating to the thicker region and thus demonstrated lower mobility^30^, which increases the probability to form the inclined twin boundary. In addition, previous simulation results^31,32^ indicated that the Peierls stress of the screw partial is much higher than that of the edge partial. Furthermore, the prominent mobility difference between screw and edge partials is supposed to contribute to the formation of the curvy dislocation lines on the inclined twin boundary.

**Fig. 4:**
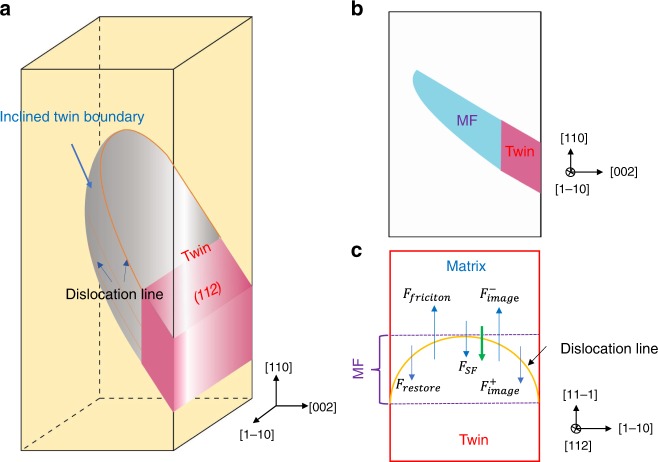
Schematic of the inclined twin boundary (Moiré fringes (MFs) region) and associated detwinning in the nanocrystal. **a** The three-dimension twin structure with the inclined twin boundary in the nanocrystal. The curved dislocation lines are marked by the orange line on the twinning plane (112) and the inclined twin boundary is marked by the gray hook surface. **b** The front view of MF along [1-10]. MF region—the projection of the inclined twin boundary region and the fully grown twin are marked by the blue and red polygons, respectively. **c** One selected twinning plane in the twin. One individual curved twinning partial on the twinning plane (112), mainly suffering four forces, the restoring force $F_{{\mathrm{{restore}}}}$, the stacking-fault force $F_{{\mathrm{{SF}}}}$, the positive and negative image forces, $F_{{\mathrm{{image}}}}^ +$ and $F_{{\mathrm{{image}}}}^ -$, respectively, and the friction force $F_{{\mathrm{{friction}}}}$ under unloading. The detwinning direction is indicated by the bold green arrow.

The instability of deformation twins and the high interface energy of the inclined twin boundary likely originate from the curved twinning partials in Bcc crystals. As shown in Fig. 4a, a group of curved twinning partials pile on parallel twinning planes (marked with (112)) in the nanocrystal due to the partial penetration of the partial dislocation, forming a 3D inclined twin boundary (the gray hook surface) and thus the corresponding MF region (cyan region in Fig. 4b) when viewed along the electron beam direction of [1-10]. To understand the detwinning mechanism, we further analyzed the dynamic behavior of twinning partials quantitatively. Due to the complex morphology and the core structure of the curved twinning partial, one simplified model was developed, as schematically shown in Supplementary Figs. 8C and 9A. Each 1/6[11-1] twinning partial was treated as a half-circle dislocation loop subject to four types of forces when external forces are absent, i.e., the restoring force $F_{{\mathrm{{restore}}}}$ due to the curvature of the dislocation line, $F_{{\mathrm{{SF}}}}$ due to the stacking-fault formation in crystals^33^, the positive and negative image forces, $F_{{\mathrm{{image}}}}^ +$ and $F_{{\mathrm{{image}}}}^ -$, respectively, originating from the opposite surfaces, and the friction force $F_{{\mathrm{{friction}}}}$ due to the lattice resistance, as shown in Fig. 4c and Supplementary Fig. 9A. $F_{{\mathrm{{restore}}}}$ reflects the strain induced by the curvature of the dislocation line and thus the elevated energy of the inclined twin boundary, whereas $F_{{\mathrm{{SF}}}}$ for one individual twinning dislocation results from the existence of the stacking fault in the interior of the nanocrystal. Considering the formation of MF via the stacking of multiple twinning dislocation on different twinning planes, the drag force due to the existence of the twin with two twin boundaries is $2F_{{\mathrm{{twin}}}\,{\mathrm{{fault}}}}$ (the twin fault force)^33^. It is noteworthy that *F* is defined as the force applied per unit length of the dislocation line in our analysis. The detailed calculation can be found in the Supplementary Discussion. Based on the analytical model, there exists a critical area fraction of MF *f*_c_ above which the average net force $\bar F_{{\mathrm{{total}}}}$ on the twin is positive (Supplementary Fig. 10), providing the driving force for spontaneous detwinning. We found that except in cases where twinning partials are located extremely close to the surface (e.g., <5 nm distance to the surface, as shown in Supplementary Fig. 11), the image force is trivial compared with the twin fault force or stacking-fault force—the property that is intrinsic to materials, indicating that the detwinning phenomenon and mechanism observed in W nanocrystals could be inherent to Bcc metals regardless of specimen size. Although the assumed geometry of the dislocation line is related to the sample size in the analytical model, the curvature of the dislocation line, which determines the restoring force and influences the structure of the inclined twin boundary in the actual materials is closely associated with the mobility of dislocations, especially for Bcc materials. Given that in bulk materials, the driving force for detwinning is mainly determined by (2$F_{{\mathrm{{twin}}}\,{\mathrm{{fault}}}}$ + *F*_restore_ − *F*_friction_), the detwinning behavior in Bcc metals should be material dependent, and those with relatively high stacking-fault and twin-boundary energies are more likely to demonstrate low stability in deformation twins.

The unstable twin structure can significantly influence the mechanical properties of Bcc metals, especially in small-sized Bcc metals where deformation twinning becomes a competing deformation mode against dislocation plasticity due to their comparable activation stresses^9,34,35^. Massive inclined twin boundaries with high interface energy formed during deformation make it difficult to induce continuous and steady twinning networks in Bcc metals. This also gives little chances to induce secondary twinning. Moreover, the inclined twin boundary as the high-energy interface provides opportunities to accumulate the mechanical energy during plastic deformation and twinning, which, followed by subsequent self-detwinning, makes small-sized Bcc metals potentially applicable for microdevices with the ability of storage and release of mechanical energy^36^ with considerable conversion efficiency. Besides, the excellent pseudoelasticity enabled by unstable twin is different from that by phase transformation^37,38^ or surface diffusion^39,40^, which makes materials recoverable to the initial structure even when they are deformed over the elastic limit^41^. In addition, self-detwinning in Bcc metals helps remove the plastic strain and may heal the “wound” induced by twinning. Consecutive twinning and detwinning during cyclic loadings might provide the pathway to bear the fatigue deformation and improve the fatigue life of Bcc metals^42^. These findings provide assistance for making Bcc nanomaterials with magnificent properties applicable in micro-electro-mechanical systems.

In conclusion, unstable twin in Bcc nanocrystals was revealed by capturing atomic-scale self-detwinning process through in-situ TEM experiments. In addition, the intrinsic instability of twin in Bcc metals is related to the inclined twin boundary with high interfacial energy that provides the driving force for spontaneous detwinning. The formation of inclined twin boundaries might be contributed to the low mobility of twinning partials in Bcc metals and the geometry of the twinning grain. The high proportion of inclined twin boundaries would facilitate complete detwinning. These findings offer new insights for understanding deformation twinning in Bcc metals, as well as guidelines for processing new structural and functional materials. For unstable twin structures, adding appropriate alloy elements may lower the interface energy and stabilize the twin and dislocation structure^43^, which provides possibilities to improve properties of Bcc metals by implanting high-density nanotwins.

## Methods

### Materials and in-situ experiment

Tungsten polycrystalline rods with diameter 0.013 inch (bought from ESPI Metal) used in the experiment and the metal purity is 99.98 wt.%. In-situ deformation tests were operated on a Nanofactory scanning tunneling microscopy inside a FEI Titian 80-300Kv TEM. The nanocrystal preparation method is referred in ref. ^9^, through welding two nano-tips together to form a nanocrystal bridge. When fracture occurred on the boundary between the nanocrystal and the substrate, as well as of the connection site, there is only physical contact and no chemical bonding between the indenter and the nanocrystal in the later compression loading, making the indenter touch the nanocrystal again. The strain rate is controlled by adjusting the displacement rate of the probe side with a piezo-manipulator and a common rate is about 10^−3^ s^−1^.

## Supplementary information

Supplementary Information

Description of Additional Supplementary Files

Supplementary Movie 1

Supplementary Movie 2

Supplementary Movie 3

## Data Availability

All data needed to evaluate the conclusions in the paper are present in the paper and/or the Supplementary Information.
