# Supplementary material for: Unstable twin in body-centered cubic tungsten nanocrystals
Source: Nat Commun. 2020 May 19;11:2497. doi: 10.1038/s41467-020-16349-8 (PMC7237484; doi:10.1038/s41467-020-16349-8)
Supplement: Supplementary file 1 — Supplementary Information [file 41467_2020_16349_MOESM1_ESM.pdf]

Supplementary Information

for

Unstable twin in body-centered cubic tungsten nanocrystals

Wang et al.

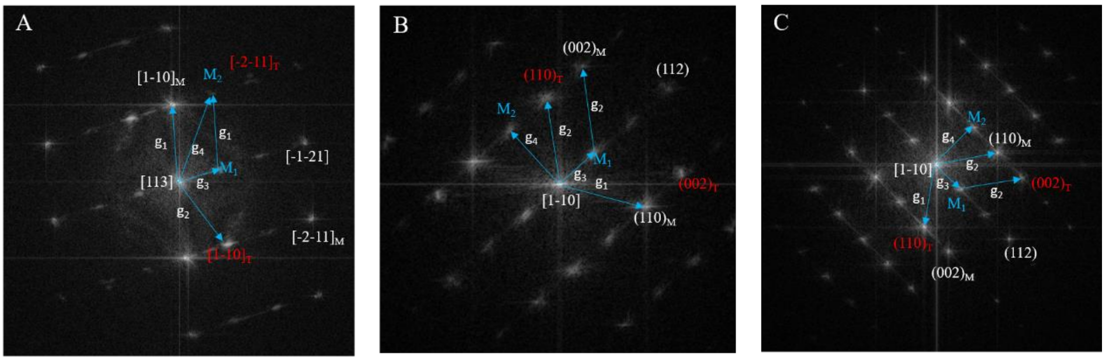

$g_3(M_1)$: g_1+g_2 | $g_3(M_1)$: g_1+g_2 | $g_3(M_1)$: g_1+g_2

$g_4(M_2)$: g_1+g_3 | $g_4(M_2)$: g_2-g_3 | $g_4(M_2)$: g_2-g_3

Supplementary Figure 1. Fast Fourier Transform (FFT) pattern analysis of the Moiré Fringes region in deformation twins of tungsten. (A) FFT pattern analysis of the Moiré Fringes region in the deformation twin showed in Figure 1 along with [113] zone axis. (B) FFT pattern analysis of the Moiré Fringes region in the deformation twin shown in Figure 2A. (C) FFT pattern analysis of the Moiré Fringes in the deformation twin shown in Figure 2B.

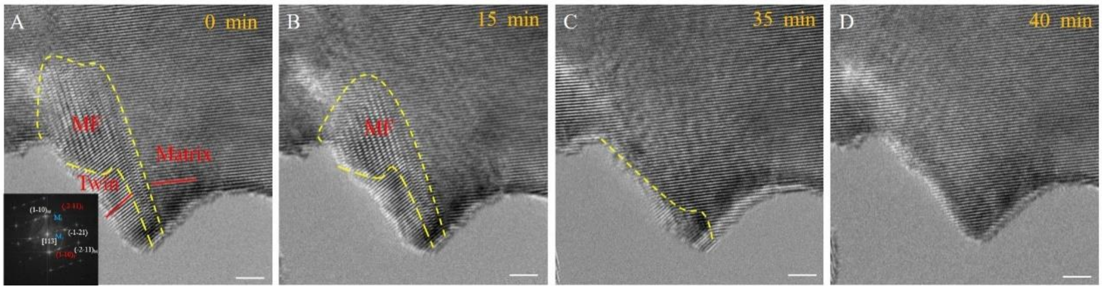

Supplementary Figure 2. Spontaneous detwinning in the twin with a high proportion of the inclined twin boundary under beam-blanked condition. (A) The deformation twin with a high proportion of Moiré Fringes. Fast Fourier Transform pattern is inserted in the figure. (B) The twin after 15 minutes under beam-blanked condition. (C) The twin after 35 minutes under beam-blanked condition. (D) The twin disappeared after 40 minutes under beam-blanked condition. Scale bar 2 nm.

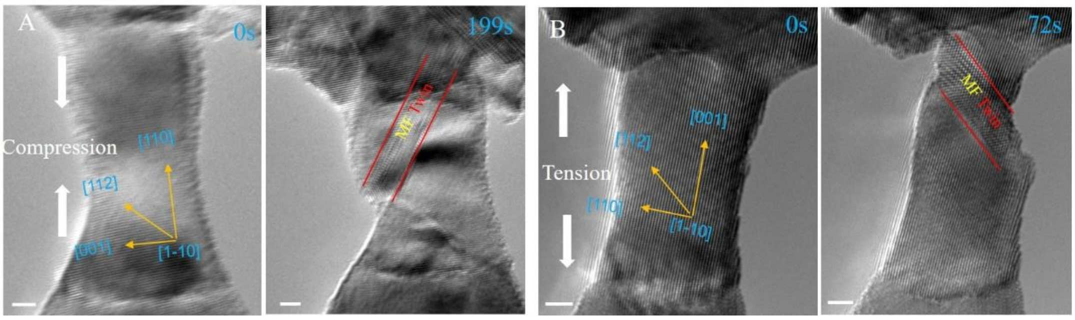

Supplementary Figure 3. Moiré Fringes forming during twinning process under compression and tension loading. (A) Compression loading. (B) Tension loading. Scale bar 2 nm.

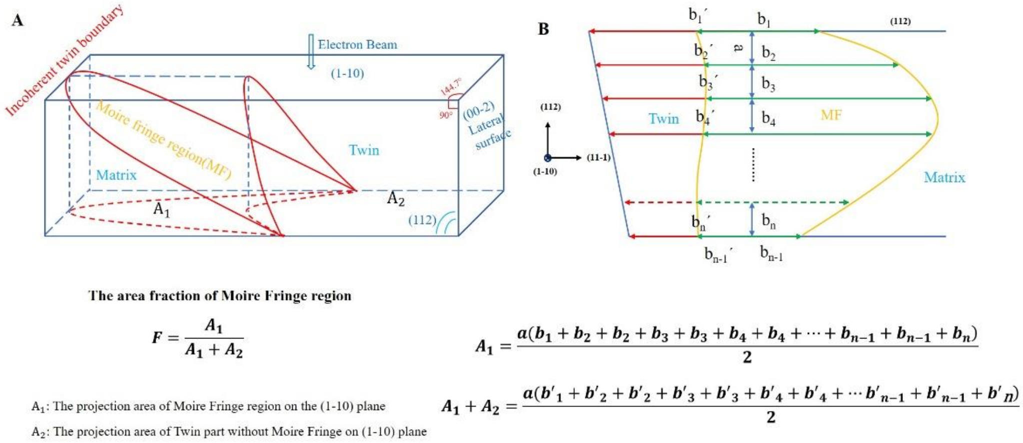

Supplementary Figure 4. Schematic of the calculation of the area fraction of Moiré Fringes (MF) region in the twin. (A) The three-dimension diagram of the twin with MF. (B) The projection of the twin with MF along the beam direction. (A_2+A_1) represents the projection area of the whole twin on the (1-10) plane ([1-10] is the view direction of TEM observation). A_1 and A_2 represent the projection areas of Moiré Fringes region and the twin part without Moiré Fringes on (1-10) plane. Due to the curved boundary of Moiré Fringes, the region could be subdivided into many small trapezoids with the same height value a. b_1', b_2', …, b_n' represent the base lengths (marked by red arrows in Supplementary Figure 4B) of trapezoids in the whole twin and b_1, b_2, …, b_n represent the base lengths (marked by green arrows in Supplementary Figure 4B) of trapezoids in Moiré Fringes region. The projection areas of the twin and Moiré Fringe region could be approximately calculated by the summation of all trapezoid areas respectively, as shown by inserted equations in Supplementary Figure 4.

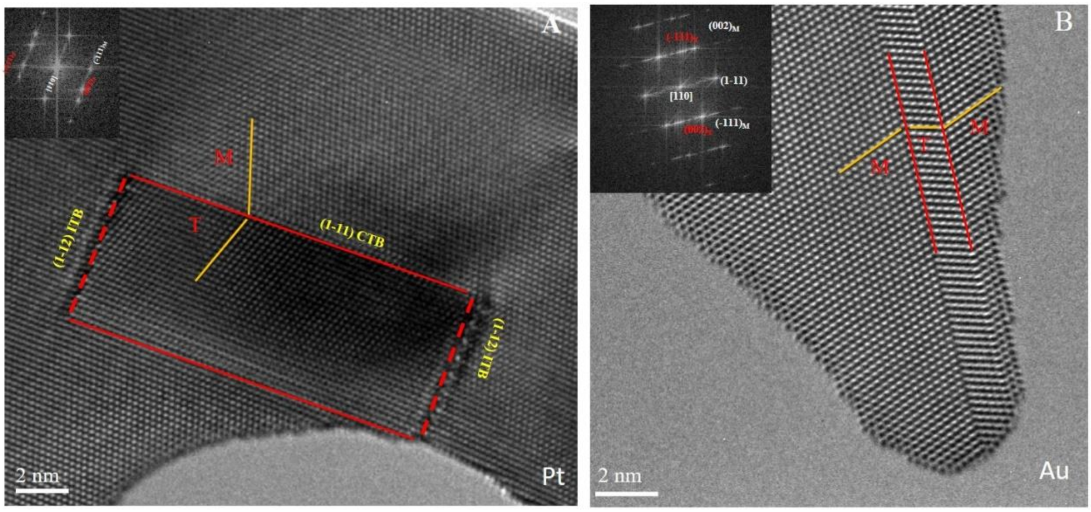

Supplementary Figure 5. Representative deformation twins in Face-centered cubic platinum (A) and gold (B). Fast Fourier Transform patterns proving the twin structure are inserted in the figure.

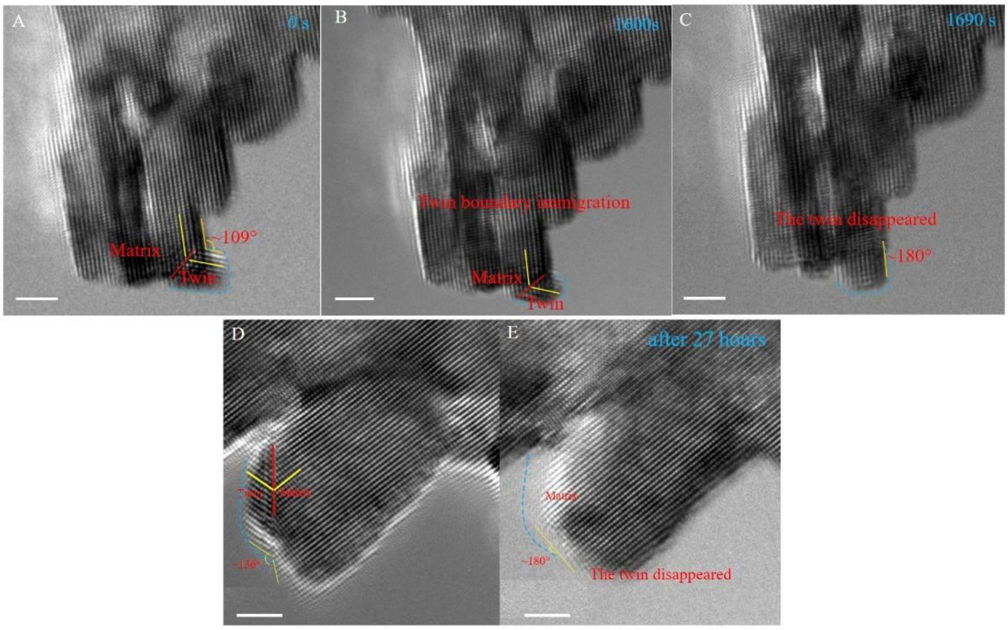

Supplementary Figure 6. Spontaneous detwinning of a small twin near the surface of W nanocrystal. (A-C) The detwinning process of one small twin near the surface. (D-E) One small twin disappeared after 27 hours under beam-blanked condition. Scale bar 2 nm.

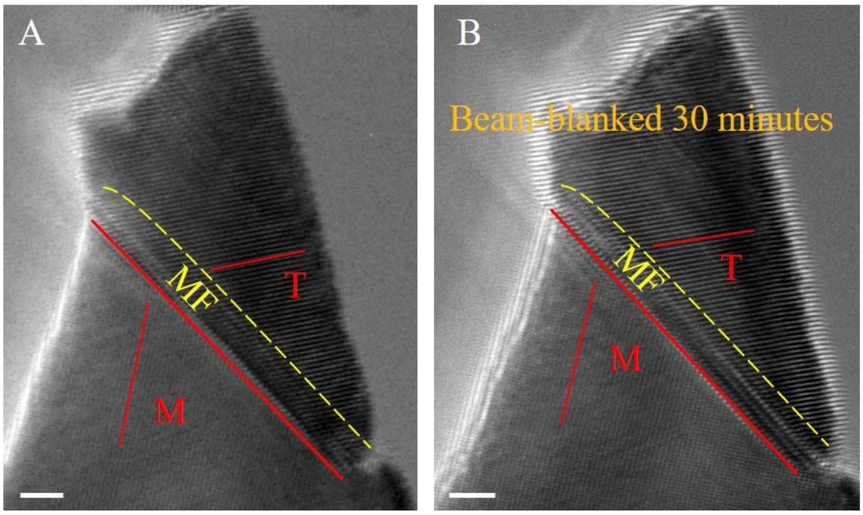

Supplementary Figure 7. Stable twin with a low fraction of Moiré fringes (MF) region in W nanocrystal. Scale bar 2 nm. (A) The deformation twin with a low fraction of MF. (B) The twin after 30 minutes under beam-blanked condition.

Supplementary Discussion on detwinning mechanism

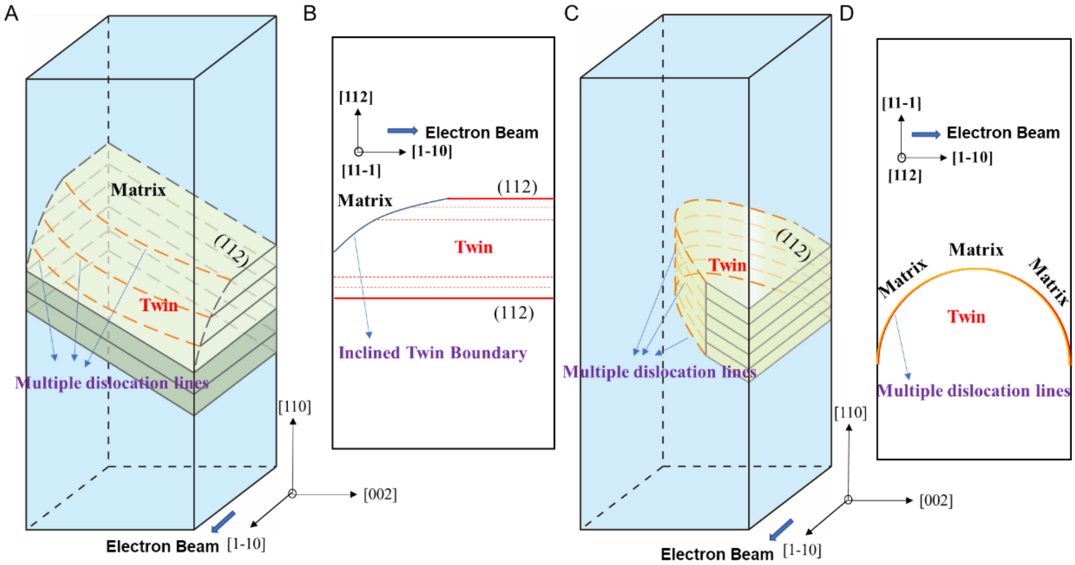

Supplementary Figure 8. The stacking of multiple twinning dislocations on different twinning planes. (A) The formation of MF via the stacking of multiple screw-type twinning partials. (B) The view of A along [11-1] direction. (C) The formation of MF via the stacking of multiple mixed-type twinning partials, based on our model. (D) The

view of C along [112] direction. The twinning dislocations are marked by the orange lines on the twinning plane (112).

The captured Moiré fringes under TEM observation mainly resulted from the lattice overlapping between the twin and the matrix along with the view direction.[1, 2] There are two main cases for the formation of the Moiré fringes as shown in Supplementary Figure 8. The first case: the formation of MF via the stacking of screw-type twinning partials, with the different propagation distances on multiple twinning planes, as shown in Supplementary Figure 8A. The second case: mixed-type twinning partial, e.g. curved dislocation, as shown in Supplementary Figure 8C. As shown in Supplementary Figure 8A, some twinning dislocations (marked by broken orange lines) don't penetrate through the whole crystal, leading to the overlapping between the matrix and the twin along the electron beam direction (Supplementary Figure 8B). However, for case 1, because the dislocation lines are parallel to the electron beam direction, it is not practically possible to determine the positions of twinning partials. Since the uncertainty of the propagation distance of twinning partials on different twinning planes, the inclined twin boundary cannot be evaluated directly. Furthermore, it is hard to develop one accurate analytical model to decide the critical condition for detwinning. Additionally, it is difficult to describe the relationship between the area fraction of MF f and the stacking of twinning partials. Based on the above difficulties associated with case 1, we select case 2 for quantitative analysis. Based on our model shown in Supplementary Figure 8C, the Moiré fringes could be formed by the stacking of one set of curved twinning dislocations on different parallel twinning planes similar to the result shown in Figure 1. As shown in Supplementary Figure 8D, the electron beam would go through the matrix and the twin, leading to the captured MF under TEM observation. For the quantitative analysis, we assume the geometry and the propagation distance of all dislocations on different twinning planes are the same.

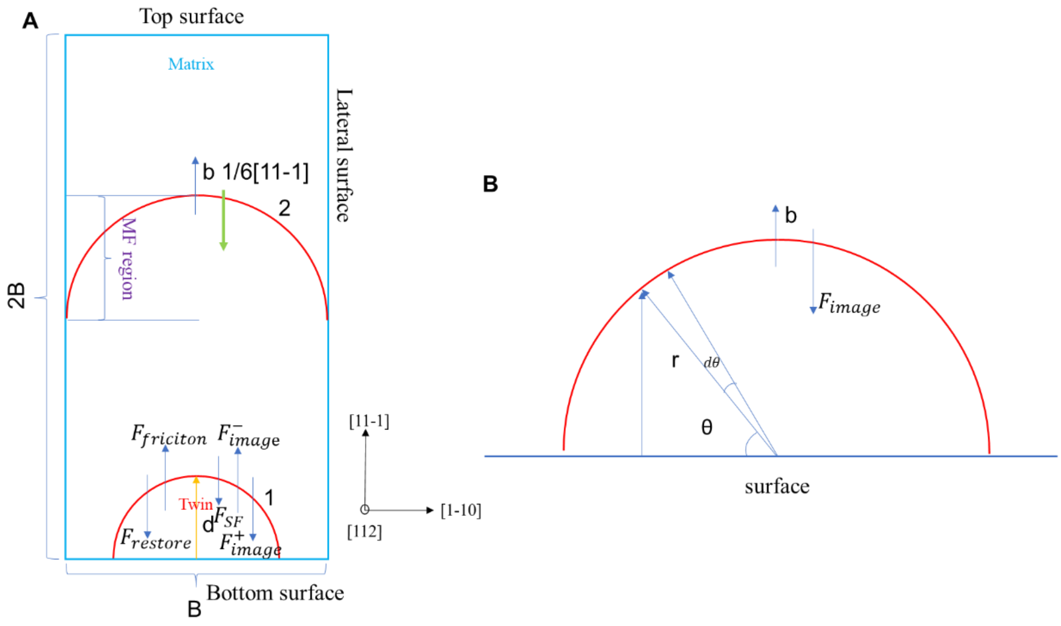

Supplementary Figure 9. (A) Schematic of the force of one individual twinning partial on the (112) twinning plane. The dislocation line is marked by the red half-loop. (B) Schematic of the image force on the half-circle loop dislocation.

For simplicity, we assumed the twinning dislocation line as one half-circle loop on one twinning plane as shown in Supplementary Figure 8C and Supplementary Figure 9A. Without external loading, there are mainly four types of forces acting on a curved twinning dislocation loop with burgers vector 1/6[11-1] on the inclined twin boundary, *i.e.* the restoring force $F_{restore}$ due to the curvature of the dislocation line, the stacking fault force F_{SF} due to the existence of the stacking fault, the positive and negative image forces, F^{+}_{image} and F^{-}_{image} respectively, originating from the opposite top and bottom surfaces, (Here, the effect from the lateral surface is ignored) and the friction force $F_{friciton}$ due to the lattice resistance (the friction force can be expressed as $F_{friction} = Peierls\ stress \cdot \mathbf{b}$. For a (112)[11-1] slip in W, $|\mathbf{b}| = 0.09$ nm, Peierls stress for edge and screw partials are 58 and 274 MPa respectively[3].). Considering the formation of Moiré Fringes via the stacking of multiple twinning dislocation on different twinning planes (Supplementary Figure 8C), the term F_{SF} would be replaced by the twin fault force $F_{twin\ fault}$.[4] In general, the twin fault force is almost half of the

stacking fault force. ($F_{twin\,fault} \approx \frac{1}{2} F_{SF}$).[5, 6] For one twin, there exists two twin boundaries and the drag force due to the existence of the twin is estimated as $2F_{twin\,fault}$. Note that F means the force applied per unit length of the dislocation line in our calculation and the directions of these four forces are indicated by arrows in Supplementary Figure 9A. The distance d is defined as the distance from the top of the dislocation line to the bottom surface. Moiré Fringes (MF region), *i.e.* the overlap region of the twinned part and the matrix, is marked by the bracket and f represents the area fraction of MF of the twin, A_m/A_T. The restoring force $F_{restore}$ can be approximately expressed by the following equation[7]:

$$F_{restore} = \frac{\alpha \mu b^2}{4\pi r} \quad (1)$$

where μ is the shear modulus, r is the radius of curvature and α is the coefficient (~2.1 for pure edge dislocation and ~6.3 for pure screw dislocation). Since F_{SF} is determined by the stacking fault energy, and it is estimated to be ~0.089 N m^-1.[8, 9, 10]

Image force for edge dislocation,[7]

$$F_{image} = \frac{\mu b^2}{4\pi l(1-\nu)} \quad (2\text{-}1)$$

Image force for screw dislocation,[7]

$$F_{image} = \frac{\mu b^2}{4\pi l} \quad (2\text{-}2)$$

For the half-circle dislocation loop, the image force as shown in Supplementary Figure 9B is

$$F_{image} = \frac{\int_0^{\pi} (\mu b\hat{\ }2\ rd\theta\ sin\theta)/4\pi r sin\theta(1-\nu) + \int_0^{\pi} (\mu b\hat{\ }2\ rd\theta\ cos\theta)/4\pi r sin\theta}{\pi r} \quad (3)$$

where l is the distance from the surface, v is the Poisson ratio, r is the radius and θ is the angle. The total force F_{total} acting on the individual dislocation line on one twinning plane is expressed as:

$$F_{total} = F_{restore} + F_{image}^{+} + F_{SF} - F_{friction} - F_{image}^{-} \quad (4)$$

Considering the stacking of multiple twinning dislocation on different twinning planes (Supplementary Figure 8C), the average net force for the twin with N layers can be expressed as:

$$\bar{F}_{total} = \frac{N(F_{restore}+F^{+}_{image}-F_{friction}-F^{-}_{image})+2F_{twin\,fault}}{N} = F_{restore} + F^{+}_{image} - F_{friction} - F^{-}_{image} + \frac{2F_{twin\,fault}}{N} \quad (5)$$

Two cases are considered in Supplementary Figure 9A as follows:

Case 1: $0 < d \leq {}^{B}/_{2}$. There is one assumption that the twinning partial keeps as a half-circle loop to propagate, the area fraction of MF *f =100%*.

Case 2: ${}^{B}/_{2} < d \leq 2\mathrm{B}$ where the assumption that the dislocation would keep as the original half-circle loop to move forward is made and the area fraction of MF *f = B/2d*. As case 2 is developed, the balance ($\bar{F}_{total} = 0$) can be reached and f_c is determined. Based on references [5, 11], we assume that the layer number of the twin $N \geq 6$.

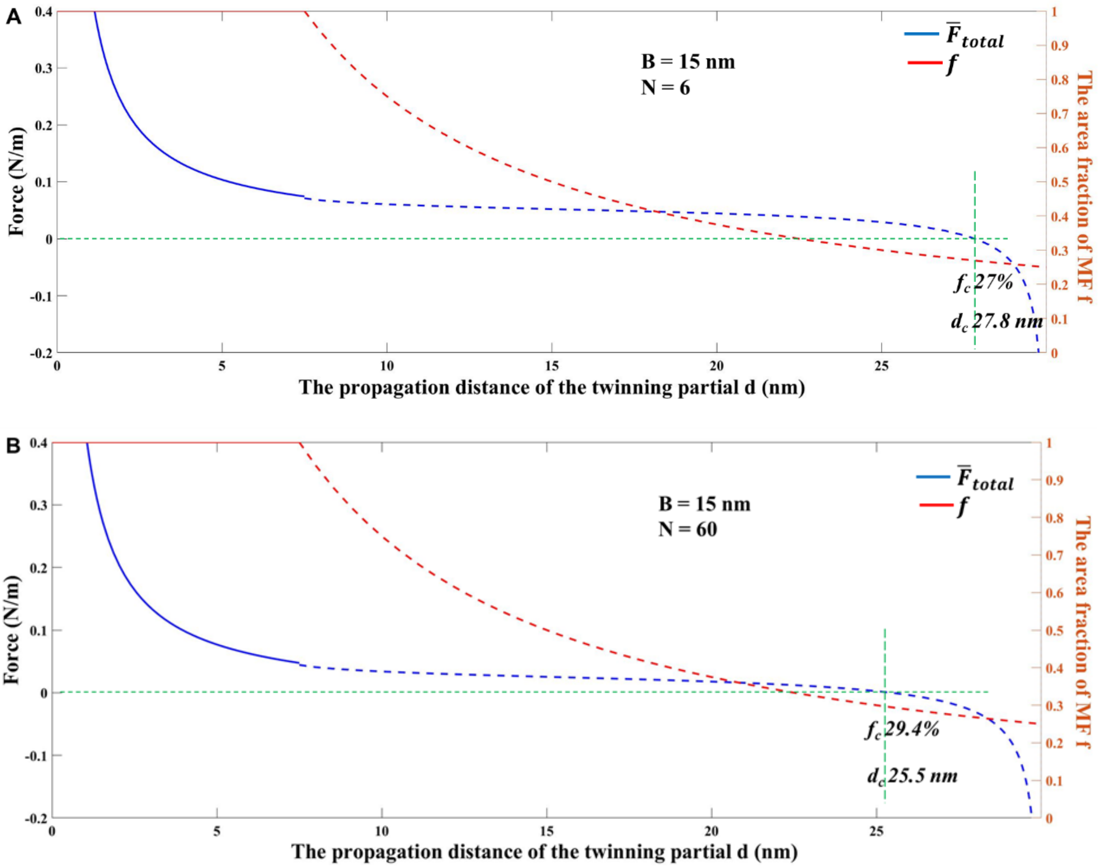

Supplementary Figure 10. (A) Multiple twinning planes, $N = 6$. (B) Multiple twinning planes, $N = 60$. The average net force $\bar{F}_{total}$ (blue line) on twinning dislocation line and the area fraction of the Moiré Fringes *f* (red line) to the propagation distance of the twining partial *d*. (*B* =15 nm, *μ* = 188 GPa in W) The solid line represents

case 1 and the broken line represents case 2.

The average net force $\bar{F}_{total}$ on the twin and the area fraction of MF f were computed based on Supplementary Equation 5 as shown in Supplementary Figure 10 where sample thickness B and width of the sample are 15 and 30 nm and setting N = 6, 60. As can be found in Supplementary Figure 10, $\bar{F}_{total}$ and f drop as twinning dislocation develops with the advancement of distance d. When d advances to 27.8 nm (Supplementary Figure 10A), the net force $\bar{F}_{total}$ on the twin becomes zero, which is the critical condition for detwinning. Then the critical area fraction of the MF f_c is determined based on zero $\bar{F}_{total}$ at d_c. In Supplementary Figure 10, when $d < d_c$, or $f > f_c$ (cases 1 and 2), the detwinning would occur spontaneously. When $d > d_c$, or $f < f_c$, the detwinning would not occur and the twin could keep stable. Based on the above analysis, the twinning and detwinning processes are partly associated with the sample size or image force. Furthermore, the effect of the sample size on the critical value f_c were evaluated by changing the parameter B (sample size) and the analysis result shows f_c drops as the sample size increases.

One is found that the predicted value of f_c for the spontaneous detwinning is much lower than the value (~ 60%) in the experiment. This difference can be ascribed to the following reasons. The first reason might be the neglect of the complex interaction between twinning partials in multiple (112) planes. Moreover, one simple assumption that the twinning partial keeps as the half-circle loop to propagate in case 2 is applied. Actually, the dislocation line may have higher curvature (higher restoring force and thus higher driving force for detwinning) rather than the half-circle loop due to the huge mobility difference between screw and edge dislocations and thus f_c would get larger. Another reason might be the underestimation of the lattice friction according to the simulation result. Our experiment indicated that twinning partials were hard to fully penetrate the crystal in the Bcc nanocrystals, while either screw or edge partial dislocation shows relatively high mobility in simulation,[5, 12] compared to the experimental observation. The potentials used for Bcc crystals in the simulation has a significant effect on the theoretical prediction, which leads to huge differences in the

lattice resistance results.[3]

It should be noted that the twin may nucleate from other surface sites and start as the formation of screw twinning partials. The screw part may dominate the dislocation type in this curved dislocation line, as shown in Supplementary Figure 8A. Owing to the dislocation line perpendicular to the view direction [1-10], for screw dislocation, it is difficult to find the propagation distance and thus hard to evaluate the image force. The complicated geometry of this case makes it hard to do the quantitative analysis as before. But it is conceivable that detwinning is still dominated by the twin fault force or stacking fault force and there also would exist one critical point as discussed before that the dislocation tends to move reversely with a certain fraction of MF.

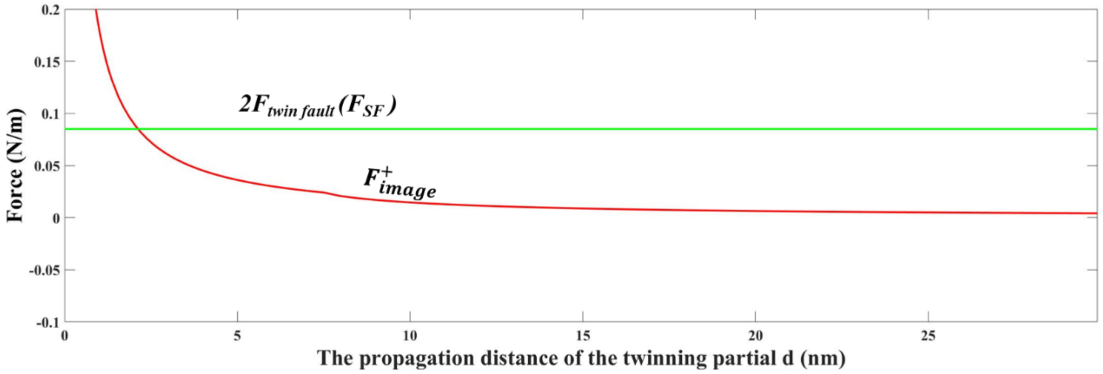

Supplementary Figure 11. Functions of $2F_{twin\,fault}$ (F_{SF}) and the positive image force F^{+}_{image} with the propagation distance of the twinning partial *d*. (*B* = 15 nm).

Supplementary References

1. Meadows D, Johnson W, Allen J. Generation of surface contours by moiré patterns. *Applied optics* **9**, 942-947 (1970).

2. Williams DB, Carter CB. The transmission electron microscope. In: *Transmission electron microscopy*). Springer (1996).

3. Ostapovets A, Paidar V. Evaluation of the Peierls stress for boundary dislocations. *The*

Physics of Metals and Metallography **111**, 229-235 (2011).

4. Hsia KJ, Suo Z, Yang W. Cleavage due to dislocation confinement in layered materials. *Journal of the Mechanics and Physics of Solids* **42**, 877-896 (1994).

5. Ogata S, Li J, Yip S. Energy landscape of deformation twinning in bcc and fcc metals. *Physical Review B* **71**, 224102 (2005).

6. Zhu YT*, et al.* Nucleation and growth of deformation twins in nanocrystalline aluminum. *Applied Physics Letters* **85**, 5049-5051 (2004).

7. Hirth JP, Lothe J. Theory of dislocations. (1982).

8. Wasilewski R. BCC stacking fault energies. *Scripta Metallurgica* **1**, 45-47 (1967).

9. Smith D, Bowkett K. The analysis of field-ion micrographs: Stacking faults in tungsten. *Philosophical Magazine* **18**, 1219-1233 (1968).

10. Vitek V. Multilayer stacking faults and twins on {211} planes in bcc metals. *Scripta Metallurgica* **4**, 725-732 (1970).

11. Ogata S, Li J, Yip S. Twinning pathway in BCC molybdenum. *EPL (Europhysics Letters)* **68**, 405 (2004).

12. Wang J, Zeng Z, Weinberger CR, Zhang Z, Zhu T, Mao SX. In situ atomic-scale observation of twinning-dominated deformation in nanoscale body-centred cubic tungsten. *Nature materials* **14**, 594 (2015).
