# Supplementary material for: Unstable twin in body-centered cubic tungsten nanocrystals
Source: Nat Commun. 2020 May 19;11:2497. doi: 10.1038/s41467-020-16349-8 (PMC7237484; doi:10.1038/s41467-020-16349-8)
Supplement: Supplementary file 2 — Description of Additional Supplementary Files [file 41467_2020_16349_MOESM2_ESM.docx]

Description of Additional Supplementary Files

**Supplementary Movie 1:** Self-detwinning in the deformation twin with a high proportion of the inclined twin boundary.

**Supplementary Movie 2:** Forming Moiré Fringes during twinning under compression.

**Supplementary Movie 3:** Forming Moiré Fringes during twinning under tension.
